# Topical Treatment with Tripeptide and Hexapeptide Following Body-Contouring Procedures Improved Patient-Reported Recovery Outcomes

**DOI:** 10.1093/asjof/ojz037

**Published:** 2020-01-14

**Authors:** Manfred M Kubler, Michael Sheehan, Laurie A Casas

As aesthetic surgeons we strive to optimize the patient experience and their satisfaction following elective nonsurgical, minimally invasive, and invasive body-contouring procedures. Specifically, patients desire safe, effective procedures with minimal recovery and downtime. With those goals in mind, we undertook an observational and patient-reported outcome measure (PROM) pilot feasibility study in a consecutive series of 15 patients (Cohort 1) to see whether an application of a topical body treatment, TransFORM Body Treatment with TriHex Technology (Alastin Skincare, Carlsbad, CA), could impact postprocedural pain, swelling, ecchymosis, and induration in patients undergoing body contouring procedures. These 15 patients enrolled in this study between September 2018 and completed the study by December 2018. This cohort consisted of 13 females and 2 males with a mean age of 45 years (range, 26-71 years) and a mean BMI of 22.6 (range, 18.1-24.2). This group was compared with a control group which consisted of a cohort of a series of 14 consecutive patients (Cohort 2) who underwent similar body contouring procedures without topical application of the product. These patients were entered in the study between July 2018 and August 2018, and completed the study by November 2018. This cohort consisted of 12 females and 2 males with a mean age of 44 years (range, 29-67 years) and a mean BMI of 23.1 (range, 18.8-25.1).

The topical body treatment with TriHex Technology includes peptides and active agents that have demonstrated efficacy in aiding lipid droplet dissolution. It is hypothesized that the fatty acids elaborated from lipid droplets released with the manipulation of fat tissue (bovie, cryolipolysis, liposuction, etc.) cause localized areas of inflammation and induration—thus more efficient removal may translate into hastened healing with decreased skin induration in the affected areas.^1^ Two observers (L.A.C., lead author and ABMS certified Plastic Surgeon, and M.S., co-author and Plastic Surgery PA-C) compared and evaluated specific recovery outcomes including patient reported pain, ecchymosis, induration, palpable soft tissue fibrosis based on observer physical exam, by utilizing 2-dimensional photographic data and EHR documentation. The visual analog scale (VAS) as a PROM for pain was administered in person to the patient by the same Medical Assistant (J.S.—not an author or observer) at each follow-up visit (Weeks 1, 2, 3, 4, 6-8 weeks, and 3-4 months). PROM of ecchymosis, swelling, and palpable soft tissue fibrosis using the scale None, Mild, Moderate, and Severe was documented by the two observers (L.A.C. and M.S.) at each postoperative interval. Each cohort of patients, Cohort 1-treated with topical product and the control group and Cohort 2-not treated with topical application of the product, underwent similar surgical body contouring procedures which included abdominoplasty, mini-abdominoplasty with liposuction of flanks and upper abdomen, bilateral mastopexy, and bilateral breast reduction in combination with liposuction to the bilateral axilla and upper abdomen. Each cohort underwent similar minimally invasive procedures which included laser liposuction procedures of the upper abdomen, bilateral back, hips, outer thighs, and inner thighs. The nonsurgical procedure performed in both cohorts was cryolipolysis. The total number of procedures in each cohort was 59. Follow-up visits were scheduled at Weeks 1, 2, 3, 4, 6-8 weeks, and 3-4 months post-procedure. This study was conducted in accordance with the WMA Declaration of Helsinki statement of ethical principles for medical research involving human subjects, including research on identifiable human material and data. Full informed written consent was acquired from each subject in the study.

Using the body treatment with TriHex Technology twice daily over the entire treated areas for 6 weeks post-procedure showed positive results by all measures. According to observers (L.A.C. and M.S.) and PROM, there was less postprocedural swelling, less pain using the Visual Analog Pain Scale, and less palpable discomfort in the patients utilizing the topical body treatment (Cohort 1) in all procedural categories when compared with the control group (Cohort 2) that did not use the topical product post-procedure. By 6 weeks, rapid resolution of the soft tissue induration and fibrosis was noted in the treated subjects, unlike the control group who experienced persistent palpable and visible soft tissue changes out to 4 months. In the surgical and minimally invasive topical treated patients in Cohort 1, the skin quality (turgor, color, and tone) was visibly and palpably improved at each follow-up visits compared with the control group (Cohort 2), who underwent similar procedures but did not use the topical application of the product postoperatively. No adverse events were reported from utilizing the topical body treatment.

A typical age, procedure, and BMI-matched representative comparison of the 2 cohorts is demonstrated in Figures 1 and 2. Figure 1A, D is the preoperative photos of the 42-year-old female patient in Cohort 2 (control group) with a BMI 28.1 who underwent abdominoplasty with liposuction of bilateral flanks and upper abdomen by the senior author (L.A.C.). The patient received *no* topical treatment. At 2 weeks post-procedure (Figure 1B,E), the patient still had persistent pain, and both swelling, and ecchymosis are still visible. At 4 months post-procedure (Figure 1C,F), swelling and persistent hyperpigmentation were noted in the areas of liposuction of the flanks. In addition, the patient felt induration and thickening of the subcutaneous tissues of the flanks and upper abdomen. Both observers (L.A.C. and M.S.) noted palpable fibrosis when the patient fully extended her arms and did a side bend and back bend. The patient noted a feeling of tightness and pulling internally when attempting these maneuvers.

**Figure 1.**
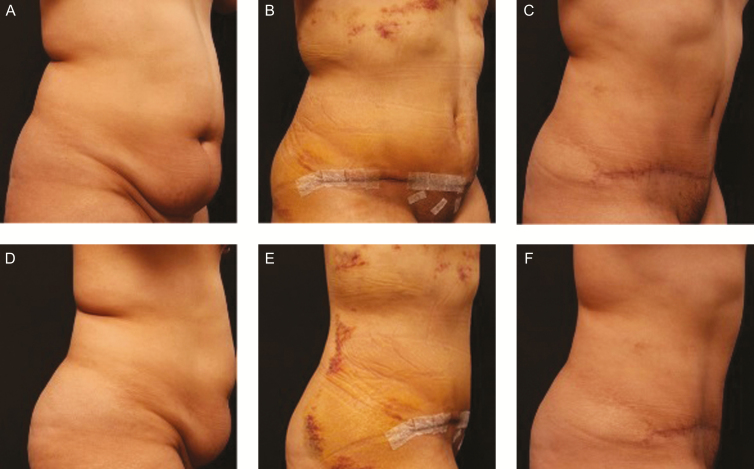
(A) Oblique and (D) lateral preoperative views of a 42-year-old female patient with a BMI of 28.1 who underwent abdominoplasty with liposuction of bilateral flanks and upper abdomen. The patient received no topical treatment (Cohort 2). (B, E) At 2 weeks post-procedure, the patient had visible swelling and ecchymosis. (C, F) At 4 months post-procedure, the patient had persistent swelling and visible hyperpigmentation in the areas of liposuction of the flanks.

**Figure 2.**
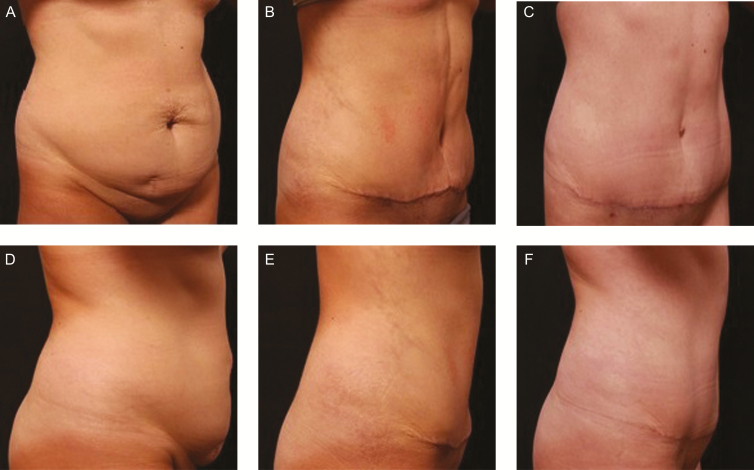
(A) Oblique and (D) lateral preoperative views of a 37-year-old female patient with a BMI of 26.7 who used the topical body treatment 2 times per day for 6 weeks over the entire procedural areas following abdominoplasty and liposuction of the flanks and upper abdomen (Cohort 1). (B, E) At 2 weeks post-procedure, the patient had complete resolution of ecchymosis. (C, F) At 4 months post-procedure, the patient did not have any signs of swelling or hyperpigmentation in the surgical areas.

In contrast, the 37-year-old female patient with BMI 26.7 in Figure 2A, D (Cohort 1) was treated with the topical body treatment two times per day for 6 weeks following abdominoplasty and liposuction of the flanks and upper abdomen. Note the complete resolution of ecchymosis at week 2 (Figure 2B,E). The patient-reported less pain and discomfort throughout the follow-up period and had no palpable subcutaneous tissue induration or fibrosis by week 6. At 4 months post-procedure (Figure 2C,F), the patient did not feel tightness or a pulling feeling in the upper abdomen or flanks when performing full arm extension and side bends. In addition, and at 4 months post-procedure, both observers (L.A.C. and M.S.) noted the absence of palpable fibrosis in the abdomen and flank areas when the patient fully extended her arms and did a side bend and back bend.

By 6 weeks post-procedure, all patients in Cohort 1 that used the topical body treatment with TriHex Technology, twice daily for 6 weeks reported less pain, discomfort, swelling and ecchymosis than the age- and the procedure-matched patients in the control group (Cohort 2). The two observers (L.A.C. and M.S.) noted a very rapid decrease in visible signs of swelling, induration, and ecchymosis, palpable subcutaneous fibrosis as well as rapid improvement in skin quality, texture, and color in all the topically treated patients (Cohort 1) at each post-procedure interval when compared with the control cohort of patients (Cohort 2).

The key ingredients of the TriHex Technology have shown histologic evidence of remodeled extracellular matrix with regenerated collagen and elastin. Facilitated through a liposome (LipoDRONE) delivery system, the actives get immediately deposited into the base of the hair follicle where this reservoir continually delivers product to the dermal white and then subcutaneous white adipose tissue.^1-3^ The hexapeptide-11 component has demonstrated in gene expression studies to accelerate (up-regulate) the process of autophagy, encouraging lipid droplet breakdown and in vitro modeling shows macrophage recruitment to damaged fat cells with in vivo trials confirming increased and hastened fat volume reduction.^1,4^ These positive clinical results in both patient-reported outcomes may relate to the optimized and hastened absorption of fatty acid particles related to the lipid droplet release seen with surgery involving adipose tissue. Fatty acid excess may provoke inflammation through the formation of “inflammasomes,” a molecular complex that results in the release of inflammatory mediators such as interleukin-1 (Il-1β).^5^ This may translate to decreased induration and the rapid dissolution of these lipid droplet particles resulting in more hastened resolution of this localized inflammation.

In summary, a noticeable reduction in postprocedural soft tissue changes and improved patient-reported recovery outcomes was evident at 2, 4, 6, and 24 weeks in a prospective consecutive series of patients (Cohort 1) that used a topical body treatment twice daily following their procedures, compared with a consecutive age- and procedure-matched Cohort 2 (control group) who did not use the topical treatment. Specifically, the patients in Cohort 1 who used the topical body treatment reported less postprocedural swelling, pain, and discomfort. In addition, the observers (L.A.C. and M.S.) noted accelerated reduction of induration and fibrosis in the soft tissue, and improved skin quality (turgor, color, and tone) both visually and palpably.

These patient-reported outcomes and observations demonstrated an improved patient experience and warrant a blinded prospective split body-controlled study to truly evaluate this concept of improved post-procedure experience.

## References

[1] Widgerow A, Kilmer S, Garruto J, Stevens W Non-surgical fat reduction and topical modulation of adipose tissue physiology. J Drugs Dermatol. 2019;18(4):375-380. 31012732

[2] Jain SK, Verma A, Jain A, Hurkat P Transfollicular drug delivery: current perspectives. Res Rep Transdermal Drug Deliv. 2016;5:1-17.

[3] Verma DD, Verma S, Blume G, Fahr A Particle size of liposomes influences dermal delivery of substances into skin. Int J Pharm. 2003;258(1-2):141-51. 12753761 10.1016/s0378-5173(03)00183-2

[4] Widgerow A, Moradi A, Poehler J A double-blind randomized controlled trial evaluating the efficacy and tolerability of a topical body treatment in combination with cryolipolysis procedures. J Drugs Dermatol. 2019;18(4): 342-348. 31012562

[5] Karasawa T, Kawashima A, Usui-Kawanishi F, et al. Saturated fatty acids undergo intracellular crystallization and activate the NLRP3 inflammasome in macrophages. Arterioscler Thromb Vasc Biol. 2018;38(4):744-756. 29437575 10.1161/ATVBAHA.117.310581

